# Measurement properties from the Brazilian Portuguese version of the QUIP-RS

**DOI:** 10.1038/s41531-020-0108-2

**Published:** 2020-02-13

**Authors:** Daniela Freitas Guerra, Ana Elisa Lemos Silva, Thiago da Silva Rocha Paz, Leandro Nogueira S. Filho, Luiz Felipe Vasconcellos, Vera Lucia Santos de Britto, Silvana Allodi, Daniel Weintraub, Alessandra Swarowsky, Clynton Lourenço Correa

**Affiliations:** 1 Programa de Pós-Graduação em Educação Física, Universidade Federal do Rio de Janeiro, Rio de Janeiro, Brazil; 2 Instituto de Neurologia Deolindo Couto, Universidade Federal do Rio de Janeiro, Rio de Janeiro, Brazil; 3 Escola de Educação Física e Desporto, Universidade Federal do Rio de Janeiro, Rio de Janeiro, Brazil; 4 Instituto de Biofísica Carlos Chagas Filho, Universidade Federal do Rio de Janeiro, Rio de Janeiro, Brazil; 5 Department of Psychiatry, University of Pennsylvania, Philadelphia, PA USA; 6 Programa de Pós-Graduação em Fisioterapia, Universidade do Estado de Santa Catarina, Santa Catarina, Brazil

**Keywords:** Parkinson's disease, Parkinson's disease

## Abstract

Parkinson’s disease (PD) has numerous motor and non-motor symptoms. Among non-motor manifestations impulse control disorders (ICDs) stand out. ICDs include compulsions for gambling, shopping, eating, and sexual behavior, and “related disorders” such as hobbyism, simple motor activities, and dopamine dysregulation syndrome. There is no rating scale translated and adapted transculturally into Brazilian Portuguese language. Therefore, we cross-culturally adapted and investigated the measurement properties of the Brazilian version of the Questionnaire for Impulsive-Compulsive Disorders in Parkinson’s Disease-Rating Scale (QUIP-RS). Fifty-three patients participated in the study. Inter-evaluator and test–retest (patient and health professional) reliabilities (intraclass correlation coefficient) were all excellent (0.93, 0.93, and 0.99). The internal consistency was high (*α* = 0.92). The Minimal detectable change (MDC) value was 5.8 (patient) and 2.3 (health professional) points. There was a floor, but no ceiling, effect. In summary, the Brazilian version of the QUIP-RS has high reliability and content validity.

## Introduction

Impulse control disorders (ICDs) and related disorders are psychiatric symptoms often found in Parkinson’s disease (PD).^1^ The main characteristic of ICDs is the impossibility of resisting an impulse, a movement to perform a typically pleasurable activity, but that can ultimately bring great harm to the patient because of its excessive nature.^2^ ICDs include compulsive gambling, shopping, eating, and sexual behavior, and “related disorders”, including dopamine dysregulation syndrome, punding and hobbyism.^3^ These compulsions occur in 15 to 35% of the PD population and are generally associated with the use of antiparkinson drugs, especially dopamine agonists.^3,4^

Over time, ICDs can negatively affect the quality of life of these individuals, causing distress to caregivers and family members,^5^ and at times serious damage to physical health, psychosocial functioning, interpersonal relationships, and financial loss,^2^ which makes its research extremely relevant. Regarding the evaluation tools that assist clinical diagnosis, screening and monitoring of ICDs symptoms, the *Questionnaire for Impulsive-Compulsive Disorders in Parkinson’s Disease-Rating Scale* (QUIP-RS)^1^ was developed and validated for this purpose. The questionnaire aims to measure severity (frequency), point out the type of compulsion, and support the diagnosis of ICDs and related disorders in PD.

The QUIP-RS has several advantages for the clinical practice, including the comprehensibility regarding instructions, low cost, objectivity relating to content (only indicative symptoms of ICDs and related disorders are evaluated), application succinctness (only four questions), easy-to-interpret vocabulary, can be self-administered by the individual or administered by health professionals or researchers. In the Brazilian scenario, there is no measurement instrument translated, transculturally adapted, and validated for assessment of ICDs symptoms in PD. The aim of the present study was to transculturally adapt and verify measurement properties (inter-rater reliability, test–retest, content validity, internal consistency, floor and ceiling effect and minimal detectable change (MDC)) of the Brazilian Portuguese version of QUIP-RS.

## Results

Regarding the two translations and back-translation, 35 changes were made, which include: substitutions, deletions, and additions of terms. Thus, in the heading, five amendments were made in Title 2, in the first question 6, in the second question 9, in third question 5, and in fourth question 8.

In the process of transcultural adaptation, the committee of specialists, based on the analysis of the final version of the Brazilian Portuguese QUIP-RS (resulting from the linguistic validation produced by MAPI), performed 22 changes (1 in the heading, 1 in the title, 1 in the first question, 5 in the second question, 5 in the third question, and 4 in the fourth question) and five modifications in the Scoring Sheet of QUIP-RS Brazil. These changes included adjustments of verbal agreement, substitutions, additions, and deletions of terms.

### Validity of content of the Brazilian Portuguese version of QUIP-RS

In order to investigate QUIP-RS Brazil through a varied clinical vision, individuals with a diversified profile were selected, both in terms of academic qualification and the area of professional emphasis. Regarding the comprehensibility of the QUIP-RS Brazil questions, the professionals agreed in their majority with the highest scores, 3 and 4. Analyzing each item, the percentage of agreement from “sufficiently clear” to “highly clear” was distributed as follows: item 1 = 60%; item 2 = 90%; item 3 = 80%; item 4 = 60%.

Regarding the comprehensibility of the possible responses, the professionals agreed mostly between the answers “sufficiently clear” and “highly clear”. The percentage of agreement obtained by the sum of these two answers was distributed as follows: Never = 100%; Rarely = 100%; Sometimes = 70%; Often = 80%; Very often = 80%.

Regarding the comprehensibility of terms representing impulse control disorders, practitioners agreed on a greater number of responses from “sufficiently clear” to “highly clear”. Thus, the percentage of the sum of these two responses was represented as follows: Gambling = 90%; Sex = 90%; Buying = 100%; Eating = 100%; Hobbyism = 60%; Compulsive repetitive activities = 70%; Use of drugs for PD = 90%.

### Sample characterization

The study included 53 patients, four of whom were excluded due to lack of clinical data. The sample had a higher prevalence of male patients (*n* = 33) compared with women (*n* = 20), the mean age of the sample was 68.3 ± 10.9, and the time since diagnosis of PD was 6.0 ± 5.2 years. All sample characterization results are shown in Table 1.

**Table 1:** Socio-demographic and clinical characteristics of participants.

Feature	Mean ± SD or %	Range
Gender		
Men	33 (62.3%)	
Women	20 (37.7%)	
Age (years)	68.3 ± 10.9	45–89
Marital status		
Single	8 (15.1%)	
Married, divorced, or widowed	45 (84.9%)	
Schooling (years)	9.1 ± 5.3	0–22
Smoking	0 (0%)	
Family history of problems with gambling	5 (9.4%)	
Problems with gambling in the family currently	0 (0%)	
Family history of alcohol abuse	20 (37.7%)	
Alcoholism	12 (2.6%)	
Prefer not to say	1 (1.9%)	
Diagnosis of PD (years)	6.0 ± 5.2	0.02–26
Modified H & Y staging (median)	2.5	
1, 1.5, 2, 2.5 (Mild)	33 (62.3%)	
3 (Moderate)	16 (30.2%)	
4 (Severe)	4 (7.5%)	
MMSE	24.7 ± 2.9	
UPDRS III	22.0 ± 12.5	6–64
UPDRS IV	2.3 ± 2.4	0–10
Deep-brain stimulation	0 (0%)	
Medication in use		
Levodopa LEDD (mg/day)	576.1 ± 279.0	0–1142.8
LEDD dopamine agonist (mg/day)	69.81 ± 78.29	0–210
Patients on dopamine agonist	25 (47%)	
TOTAL LEDD (mg/day)	662.8 ± 317.7	0–1390.7
Use of amantadine	15 (28.3%)	
Use of antidepressant	12 (22.6%)	
Frequency for symptoms indicative of ICDs	26 (49.1%)	
Total score in Brazilian Portuguese QUIP-RS	6.8 ± 7.9	0–26

*PD* Parkinson’s disease, *MMSE* Mini-Mental State Examination, *UPDRS* Unified Parkinson’s Disease Scale, *SD* standard deviation, *LEDD* levodopa equivalent daily doses, *ICDs* impulse control disorders, *QUIP-RS* Questionnaire for Impulse-compulsive disorders in Parkinson’s Disease-Rating Scale.

### Reliability

Regarding the ICC (95% CI) values of inter-rater reliability (Table 2), all presented excellent concordances (ICC > 0.75)—ranging from 0.84 to 0.96—for the total score of each compulsion and for total ICDs and total QUIP-RS Brazil score. Regarding the values of *k* (95% CI), all had excellent concordances—ranging from 0.79 to 0.94—in each evaluated item.

**Table 2:** Specific health professional vs. patient self-evaluation comparison for the total score of each impulse control disorder, total ICD score, and total score of the QUIP-RS.

*n* = 53	Patient median (min–max)	Patient mean (SD)	Professional median (min–max)	Professional mean (SD)	ICC (IC 95%)	*k* (IC 95%)
Gambling	0 (0–4)	0.30 (0.75)	0 (0–4)	0.25 (0.70)	0.88 (0.79–0.93)	0.79 (0.56–1)
Sex	0 (0–9)	1.53 (2.20)	0 (0–9)	1.42 (2.20)	0.92 (0.87–0.96)	0.89 (0.77–1)
Buying	0 (0–7)	0.55 (1.31)	0 (0–7)	0.49 (1.25)	0.89 (0.27–0.93)	0.85 (0.68–1)
Eat	0 (0–12)	1.81 (2.84)	0 (0–12)	1.91 (3.04)	0.96 (0.93–0.98)	0.93 (0.85–1)
Hobbyism- compulsive and repetitive behaviors	0 (0–12)	1.62 (2.40)	0 (0–12)	1.30 (2.21)	0.84 (0.74–0.90)	0.83 (0.66–1)
Use of PD drugs	0 (0–8)	1.57 (2.07)	0 (0–8)	1.45 (2.06)	0.96 (0.92–0.97)	0.94 (0.86–1)
Total ICD punctuation	2 (0–17)	4.19 (5.01)	0 (0–15)	4.06 (4.88)	0.96 (0.93–0.98)	0.93 (0.87–0.99)
Total punctuation	3 (0–28)	7.38 (8.31)	0 (0–26)	6.81 (7.93)	0.93 (0.88–0.96)	0.90 (0.81–0.99)

*Min* Minimum, *max* maximum, *ICC* intraclass correlation coefficient, *CI* confidence interval, *k* kappa, *SD* standard deviation, *PD* Parkinson’s disease, *ICD* impulse control disorder, *QUIP-RS* Questionnaire for Impulse- compulsive disorders in Parkinson’s Disease-Rating Scale.

Analyzing the ICC values of the test–retest reliability of the patients (Table 3), all the items analyzed had excellent concordances (ICC > 0.75)—ranging from 0.85 to 0.99—the total score of each compulsion, total ICD score, and total QUIP-RS Brazil score.

**Table 3:** Test–retest reliability (patient) for the total score of each impulse control disorder, total ICD score, and total QUIP-RS score.

*n* = 53	Patient Median (min–max)	Patient Mean (SD)	ICC (IC 95%)
Gambling	0 (0–4)	0.30 (0.75)	0.88 (0.79–0.93)
Sex	0 (0–9)	1.53 (2.20)	0.99 (0.98–0.99)
Buying	0 (0–7)	0.55 (1.31)	0.85 (0.75–0.91)
Eat	0 (0–12)	1.81 (2.84)	0.96 (0.93–0.98)
Hobbyism-compulsive and repetitive behaviors	0 (0–12)	1.62 (2.40)	0.86 (0.76–0.92)
Use of PD drugs	0 (0–8)	1.57 (2.07)	0.95 (0.91–0.97)
Total ICD punctuation	2 (0–17)	4.19 (5.01)	0.96 (0.93–0.98)
Total punctuation	3 (0–28)	7.38 (8.31)	0.93 (0.88–0.96)

*Min* minimum, *max* maximum, *SD* standard deviation, *ICC* Intraclass correlation coefficient, *CI* confidence interval, *PD* Parkinson’s disease, *ICD* impulse control disorder, *QUIP-RS* Questionnaire for Impulse-compulsive disorders in Parkinson’s disease-Rating Scale.

Regarding the ICC, values for the test–retest reliability of the health professional (Table 4) investigated in each item of the questionnaire were found to be between 0.93 and 1, therefore considered excellent.

**Table 4:** Test–retest reliability (health professional) for the total score of each impulsive control disorder, total ICD score, and total QUIP-RS score.

*n* = 53	Professional median (min–max)	Professional mean (SD)	ICC (IC 95%)
Gambling	0 (0–4)	0.25 (0.70)	1 (1–1)
Sex	0 (0–9)	1.42 (2.20)	0.96 (0.94–0.98)
Buying	0 (0–7)	0.49 (1.25)	0.93 (0.88–0.96)
Eat	0 (0–12)	1.91 (3.04)	0.96 (0.93–0.98)
Hobbyism- compulsive and repetitive behaviors	0 (0–12)	1.30 (2.21)	1 (1–1)
Use of PD drugs	0 (0–8)	1.45 (2.06)	0.99 (0.98–0.99)
Total ICD punctuation	0 (0–15)	4.06 (4.88)	0.97 (0.94–0.98)
Total punctuation	0 (0–26)	6.81 (7.93)	0.99 (0.98–0.99)

*Min* minimum, *max* maximum, *SD* standard deviation, *ICC* intraclass correlation coefficient, *CI* confidence interval, *PD* Parkinson’s disease, *ICD* impulsive control disorder, *QUIP-RS* questionnaire for Impulse-compulsive disorders in Parkinson’s disease-Rating Scale.

### Internal consistency, floor and ceiling effect, and MDC

The internal consistency of the QUIPRS Brazil was analyzed in a subset of patients (*n* = 32) and measured between the scores of each compulsion within each item of the questionnaire. The value of the Cronbach alpha coefficient equal to (*α* = 0.92) pertinent to internal consistency, indicated high reliability.

In order to investigate the floor and ceiling effect, the total score of the questionnaire was considered, so the floor effect (score 0) was observed in 50.9% of the sample, but no ceiling effect was observed.

The MDC value established for self-administration of QUIP-RS Brazil by patients was 5.8 points. MDC established for administration of QUIP-RS Brazil by health professionals was 2.3 points.

## Discussion

Self-report questionnaires have been produced in a high amount in the scientific context, but their development is expensive, since it entails large expenditure of time and money. So, if there is no validated measuring instrument for a culture, it is recommended that researchers do the translation/adaptation/validation of previously developed questionnaires originated in another language, because this process is considered less expensive.^6^ The current research, aiming to fill the gap in the literature, investigated both the transcultural adaptation of QUIP-RS to Brazilian Portuguese and its measurement properties.

Concerning the interval reliability, the total score of the QUIP-RS Brazil obtained excellent values (ICC = 0.93), as did the total scores of each compulsion and the total ICD score (ranging from 0.84 to 0.96). The results of this reliability related to the original questionnaire also reached excellent agreement in QUIP-RS total score (ICC = 0.93); (0.9–0.97), the range of values of the QUIP-RS Brazil^1^ was similar. Regarding the inter-rater reliability results analyzed with *k*, all eight items evaluated obtained excellent agreement (0.79–0.94). For the purposes of registration, *k* was not determined in the original QUIP-RS research, as the inter-rater reliability was not investigated in the German-language QUIP-RS study.^1,7^

The total scores of the QUIP-RS Brazil test–retest reliability of the patients and the health professional achieved excellent concordances ICC = 0.93 and ICC = 0.99, respectively, which in turn were higher than test–retest reliability score of the original QUIP-RS (ICC = 0.87). All eight items evaluated (QUIP-RS Brazil) in both test–retest (patients and health care professional), obtained excellent concordances and presented the following range of values: 0.85–0.99; 0.93–1. Unlike the Brazilian version, the German version achieved excellent agreement only in three items: having sex/ICC = 0.80; eating/ICC = 0.78; and total score QUIP-RS/ICC = 0.78. In addition, the remaining five items of the German QUIP-RS had moderate agreement and ranging (ICC) from 0.57 to 0.73. Regarding the results of test–retest reliability of the original version, of the eight analyzed items, seven obtained excellent agreement (0.77–0.97), and only one obtained moderate agreement (compulsive repetitive hobby/activities/ICC = 0.68).^1,7^ Test–retest reliability with health care professionals was not investigated either in the US research (original QUIP-RS) or in the German version research.

As far as content validity results are concerned, most of the professionals’ agreement about the evaluated items was between “sufficiently clear” and “highly clear”. Nevertheless, to a lesser extent, it was pointed out by practitioners that some items in the questionnaire were “a little clear”. It is inferred that, in a general way, the professionals understood the different items that make up the questionnaire. Content validity has not been verified in the original QUIP-RS or German version.

Regarding floor and ceiling effect, the results did not show ceiling effect; however, floor effect was observed in >15% of patients. This is because 50.9% of the sample received a zero score, that is, it did not show frequency for suggestive symptoms of ICDs. The floor and ceiling effect were not examined by the original QUIP-RS survey either in Germanic research.

Possible explanations for the low total values of QUIP-RS Brazil and the floor effect (score 0 obtained in 50.9% of the sample) are: the dosage of dopamine agonist—drugs associated with ICDs—and the moderate prevalence of the use of dopamine agonists in the present study sample. In this sense, although the dosage of dopamine agonists associated with the origin of the ICDs has not been identified,^3^ it is suggested that total levodopa equivalent daily doses (LEDD) dopamine agonists (52.68 mg/day) of the Brazilian sample is not sufficient to trigger or elevate the frequency of symptoms of ICDs at high values. Thus, the mean value of dopamine agonist LEDD is low compared to the original American QUIP-RS sample (185.6 mg/day), and the value of the sample from the German version of QUIP-RS (166.43 mg/day).^1,7^ The prevalence of dopamine agonists did not reach half of the current study sample (47%), so it is suggested that this may have contributed to the low frequency of IDCs.

In addition, the patients had frequent neurologist follow-up and constant monitoring of drug therapy. Any symptoms abnormalities were reported to the neurologist responsible. Moreover, other justifications that may explain the lack of frequency of ICD-indicative symptoms in 50.9% of the sample are due to the fact that most of patients do not have the following risk factors for the development of compulsions: married, divorced, and widowed (83.7%), do not drink alcohol (73.5%), do not smoke (100%), have no family history of gambling problems (89.8%), and/or alcohol consumption (100%) did not do deep-brain stimulation (100%).^3^

The reliability analysis of the QUIP-RS Brazil was also examined through internal consistency, which is established by calculating the Cronbach alpha coefficient. Thus, it is suggested that the items of a scale have *α* Cronbach of >0.70.^8^ In the present study, the QUIP-RS Brazil items revealed an *α* = 0.92, and this indicates that there is a strong relation of each compulsion symptom punctuation with each item of the questionnaire. It is worth noting that internal consistency has not been investigated by US and German research.^1,7^

Considering that the questionnaire presents two application possibilities—self-administration by the patient and administration by the health professional—two MDCs were established for the QUIP-RS Brazilian Portuguese version. Thus, the MDC when self-administered by the patient scored 5.8 points and MDC when administered by the professional scored 2.3 points. Therefore, a change in the total score between two evaluations above or below these values, has a chance <5% of being due to random error or chance variation.^9^ Those MDCs represent 5.2% and 2%, respectively, of the total score (112) of the Brazilian Portuguese version. Therefore, the present study indicates that the questionnaire could be able to recognize even a small clinical change after the intervention. MDC was not investigated in the American research (original QUIP-RS) nor in the German research.

This study presented as a limitation the absence of the investigation of the reliability of QUIP-RS Brazilian Portuguese version with the participation of caregivers and relatives. The caregivers “contribution in this process would be interesting because they would help confirm or not the patients” compulsive attitudes in their daily lives.

## Methods

The present work is characterized as a cross-sectional and methodological study of transcultural adaptation and investigation of measurement properties carried out in two phases. The first phase consisted of the analysis and production process of the pre-final version of the QUIP-RS Brazil by a committee of experts (one linguist, two university professors, one methodologist), then the process of cross-cultural adaptation of the English language to the Brazilian Portuguese, followed by approval of the developer of the QUIP-RS (DW). The second phase consisted of administration (by the health professional) and self-administration (patient responds alone) of the Brazilian Portuguese version of QUIP-RS in patients with PD for measurement property analysis.^10^ The first phase included the translated versions, back-translation, and a final version of the QUIP-RS for Brazilian Portuguese conducted by the *Mapi Research Institute (MAPI)* method. These versions were provided by the QUIP-RS developer (DW).

The research project was submitted to the Human Ethics Research Committee of the Neurology Institute Deolindo Couto-INDC/UFRJ (Rio de Janeiro, Brazil) under terms of Resolution 466/2012 National Health Council, approved under the CAAE registry 50609315.5.0000.5261. All of the research participants were informed of the goals of the present study and after agreement signed the Term of Free and Clarified Consent based on the Declaration of Helsinki.

### Population

The study population was intentionally recruited and evaluated at INDC-UFRJ, Rio de Janeiro, and at the State University of Santa Catarina (UDESC), Brazil. Fifty-seven patients accepted to participate in this study; however, four patients were excluded due to lack of clinical data resulting in a total of 53 patients for analysis. The evaluations were carried out in the “ON” state of PD medication use, with an interval of at least 2 h and a maximum of 4 h after ingestion, always in the morning.

Inclusion criteria were: adults with a clinical diagnosis of idiopathic PD confirmed by a neurologist according to the United Kingdom PD Society Brain Bank criteria;^11^ patients under antiparkinson drug treatment; patients with stage I to IV of the Modified Hoehn & Yahr Scale;^12^ and score in the Mini-Mental State Examination (MMSE) within normality according to education level.^13^ Individuals that did not reach established scores in the MMSE were excluded from the study.

### Evaluation tools

The original QUIP-RS has four questions that assess patient-specific behaviors and impulses in the last 4 weeks, or any 4-week period at a given time.^1^ These questions include the analysis of four ICDs (gambling, shopping, eating, and sexual behavior) plus related disorders (medication use, simple motor activity, and hobbyism). QUIP-RS has an instruction sheet in which each evaluated behavior is specified in more detail. The total score of the questionnaire can range from 0 to 112 points. The higher the score, the greater the frequency and severity of symptoms indicative of ICDs.

The severity of the disease was assessed by Unified Parkinson’s Disease-Rating Scale (UPDRS)^14^ and by Modified Hoehn & Yahr Scale.^12^ For global cognitive assessment we used the Mini-Mental State Examination (MMSE).^15^ The cutoff values used were as follows: illiterates/13; low/average schooling/18; high schooling/26.^13^

In order to obtain levodopa equivalent daily dose (LEDD), the equivalent units used in the study were: 100 mg levodopa = 130 mg controlled-release levodopa = 70 mg levodopa + catechol inhibitor-O-methyl transferase (COMT inhibitor) = 1 mg pergolide = 1 mg pramipexole = 5 mg ropinirole.^16^

Considering that the QUIP-RS investigates a topic of intimate character, the health professional was careful when evaluating the patient with the questionnaire using an approach that is not in its objective aspect, that is, purely technical. The evaluation was conducted in a manner to establish a trusting relationship with the patient.

### Transcultural adaptation

The translation and cultural adaptation phases of QUIP-RS Brazil were performed based on the adapted method^10^ and with the participation of MAPI (Research Institute that has a language validation method). The first three stages were performed by MAPI, Stage I (Initial Translation), Stage II (Synthesis of Translations), and Stage III (Reverse Translation). At these stages, two qualified translators performed two translations and subsequently carried out the reconciliation (synthesis of the two translations). A back-translation was produced by a qualified translator. Stage IV (Expert Committee) and Stage V (Pre-Final Test) were performed by the authors. In stage IV, a team of professionals (one linguist, three professors) consolidated all previous versions of the QUIP-RS and produced the pre-final version of the questionnaire. In Stage V, the field pre-test of the pre-final version of the Brazilian Portuguese version of QUIP-RS was performed. The questionnaire was applied to five PD patients who were not included in the study to verify the comprehension of the items. Moreover, the last step included the developer of the scale (DW), Stage VI (submitting documentation to the developer for consideration of the adaptation process). At this stage, all documents, including the pre-final version of the Portuguese-Brazilian QUIP-RS produced by the expert committee, were presented to the developer of the original questionnaire. The QUIP-RS Brazil translations and back-translations produced by MAPI was kindly provided for current research by the original questionnaire developer.

### Measurement properties

The inter-examiner reliability was obtained by self-administration (patient completed alone) the QUIP-RS Brazil. Besides, health professional vs. patient self-evaluation data were collected in order to compare results of QUIP-RS Brazil from health professional and patients self-evaluation.^17^ There was a time difference from 20 min to 30 min between both measurements (firstly professional and subsequently patient self-evaluation). Both measurements were performed during the morning. The inter-examiner reliability as well as health professional vs. patient self-evaluation comparison were obtained at two times, the second after 1 week.^1^

Internal consistency indicates the degree of relationship between the individual items of an evaluation instrument. In the Brazilian version of the QUIP-RS, this property was measured between the score of each compulsion within each question in the questionnaire.^18^

The upper and lower effects were established by calculating the percentage (>15%) obtained by identifying the minimum and maximum scores that each patient received in the QUIP-RS Brazilian version.^19^

The MDC is an index considering the standard error of the evaluation instrument. It is important to establish the minimum variation value in the instrument score, which can be interpreted as a clinically relevant change assigned to a clinical improvement and not to a variation in measurement.^9^

In order to verify the comprehensibility of the translation of the items that make up the Brazilian Portuguese QUIP-RS through a clinical approach, a form for content validity analysis was produced. This form was composed of the QUIP-RS Brazil items, which were evaluated using a 4-point Likert scale (1 corresponds to “not clear”, 2 “a little clear”, 3 “sufficiently clear”, 4 “highly clear”).^20^

Three-hundred twenty-six health professionals were invited, of which 40 confirmed participation and 17 completed the form. From this sample, taking into consideration that five professional experts are recommended as a minimum number to verify the validity of content, the current study chose ten forms for statistical analysis.^19^ The professionals who participated in this research, physicians, and physiotherapists, belong to different areas of health care (Neurology, Medical Sciences, Neurosciences, Health Technology, Adult Neurorehabilitation, Child and Women’s Health).

### Statistical analysis

The socio-demographic and clinical characteristics of the patients were estimated through analysis using descriptive statistics. The intraclass correlation coefficient (ICC) with confidence intervals was used to test the test–retest reliability and inter-rater, calculated on the items total score of each impulse control disorder (ICD), total ICD score and total QUIP-RS Brazil score (CI) of 95%, and concordance standards of ICC < 0.40 (weak); ICC ≤ 0.75 (moderate); ICC > 0.75 (excellent).^21^

In order to obtain a more accurate assessment of inter-rater reliability, the current study used Cohen’s kappa (*k*) in the items total score of each impulsive-compulsive disorder, total ICD score, and total QUIP-RS Brazil score. The concordance standards used as reference for analysis were: 0.81–1.00 (excellent); 0.61–0.80 (substantial); 0.41–0.60 (moderate); 0.21–0.40 (considerable); 0–0.20 (slight); and poor when equal to zero.^22^

The internal consistency was verified by means of the Cronbach alpha coefficient.

To verify the content validity, the following formula (equation 1) was used:^23^

1 $$\% \,{\mathrm{agreement}} = \frac{{{\mathrm{number}}\,{\mathrm{of}}\,{\mathrm{participants}}\,{\mathrm{who}}\,{\mathrm{agreed}}\,{\times}\,100}}{{{\mathrm{total}}\,{\mathrm{number}}\,{\mathrm{of}}\,{\mathrm{participants}}}}$$

MDC was obtained using the following formula:

MDC = *Z-*score_level of confidenc_ × SD_baseline _ × *√* (*2* [*1* *–* *r*_test–retest_)], 95% CI was adopted.^9^

The results of reliability, validity as well as internal consistency were analyzed by the MedCalc 12.5.0 program and IBM SPSS Statistics 24 for Windows. For the descriptive statistics, Excel 2016 program for Windows was used.

To conclude, our results indicate that the QUIP-RS Brazilian Portuguese version presents reliability, internal consistency and content validity. The QUIP Brazilian Portuguese version has the possibility of identifying the severity of ICD symptoms in patients with PD. However, research that confirms or refutes the presence of floor effect is needed.

The translation/transcultural adaptation/validation/reliability of the Brazilian Portuguese version of the QUIP-RS opens up an innovative precedent (the first Portuguese-speaking country to investigate the measurement properties of a translated and culturally adapted version of QUIP-RS) that can be used a base not only for translation/validation/reliability of the QUIP-RS in Portuguese-speaking countries, but also provides results for a future participation in international multicenter researches that can contribute scientifically contrasting or confirming studies results with the QUIP-RS in foreign languages.

### Reporting summary

Further information on experimental design is available in the Nature Research Reporting Summary linked to this article.

## Supplementary information

Measurement properties from the Brazilian Portuguese version of the QUIP-RS

Reporting Sum

## Data Availability

The datasets generated during and/or analyzed during the current study are available from the corresponding author on reasonable request.

## References

[1] Weintraub D (2012). Questionnaire for impulsive–compulsive disorders in Parkinson’s disease rating scale. Mov. Disord..

[2] Weintraub D (2008). Review. Dopamine and impulse control disorders in Parkinson’s disease. Ann. Neurol..

[3] Weintraub D, Siderowf AD, Whetteckey J (2010). Impulse control disorders in parkinson disease. A cross–sectional study of 3090 patients. Arch. Neurol..

[4] Joutsa J, Martikainen K, Vahlberg T, Voon V, Kaasinen V (2012). Impulse control disorders and depression in Finnish patients with Parkinson’s disease. Parkinsonism Relat. Disord..

[5] Weintraub D (2009). Impulse control disorders in Parkinson’s disease: prevalence and possible risk factors. Parkinsonism Relat. Disord..

[6] Epstein J, Santo RM, Guillemin F (2015). A review of guidelines for cross-cultural adaptation of questionnaires could not bring out a consensus. J. Clin. Epidemiol..

[7] Probst CC (2014). Validation of the questionnaire for impulse control disorders in Parkinson’s disease (QUIP) and the QUIP-rating scale in a German speaking sample. J. Neurol..

[8] Lohr KN (1996). Evaluating quality-of-life and health status instruments: development of scientific review criteria. Clin. Therapeutics.

[9] Haley SM, Fragala-Pinkham MA (2006). Interpreting change scores of tests and measures used in physical therapy. Phys. Ther..

[10] Beaton DE, Bombardier C, Guillemin F, Ferraz MB (2000). Guidelines for process of cross-cultural adaptation of self-reports measures. Spine (PhilaPa 1976).

[11] Gibb WR, Lees AJ (1988). The relevance of the Lewy body to the pathogenesis of idiopathic Parkinson’s disease. J. Neurol. Neurosurg. Psychiatry.

[12] Goetz CG (2004). Movement disorder society task force report on the Hoehn and Yahr staging scale: status and recommendations. Mov. Disord..

[13] Bertolucci PH, Brucki SM, Campacci SR, Juliano Y (1994). O mini-exame do estado mental em uma população geral–Impacto da escolaridade. Arquivos de. Neuro-psiquiatria.

[14] Fahn, S. & Elton, R. L. in: *Recent Developments in Parkinson’s Disease*, 2nd edn, 153–163, 293–304 (Macmillan Healthcare Information, Florham Park, NJ, 1987).

[15] Folstein MF, Robins L, Helzer NJE (1983). The mini-mental state examination. Arch. Gen. Psychiatry.

[16] Weintraub D (2006). Association of dopamine agonist use with impulse control disorders in Parkinson disease. Arch. Neurol..

[17] Jang DH (2013). Reliability and validity of the Korean version of the manual ability classification system for children with cerebral palsy. Child.: Care Health Dev..

[18] Devitt J (1998). Testing internal consistency and construct validity during evaluation of performance in a patient simulator. Anesthesia Analgesia.

[19] Terwee CB (2007). Quality criteria were proposed for measurement properties of health status questionnaires. J. Clin. Epidemiol..

[20] Swarowsky A (2017). Cross cultural adaptations and psychometric domains of Brazilian version of PROFILE PD for Parkinson’s disease. Disabil. Rehabilitation.

[21] Fleiss JL, Levin B, Palk MC (2003). Statistical Methods for Rates and Proportions.

[22] Landis JR, Koch GG (1977). The measurement of observer agreement for categorical data. Biometrics.

[23] Tilden VP, Nelson CA, May BA (1990). Use of qualitative methods to enhance content validity. Nurs. Res..

