# Supplementary material for: Measurement properties from the Brazilian Portuguese version of the QUIP-RS
Source: NPJ Parkinsons Dis. 2020 Feb 13;6:6. doi: 10.1038/s41531-020-0108-2 (PMC7018811; doi:10.1038/s41531-020-0108-2)
Supplement: Supplementary file 1 — Measurement properties from the Brazilian Portuguese version of the QUIP-RS [file 41531_2020_108_MOESM1_ESM.pdf]

Questionário para transtornos impulsivo-compulsivos na doença de Parkinson - escala de classificação (Questionnaire for Impulsive-Compulsive Disorders in Parkinson's Disease - Rating Scale, QUIP-RS)

Informado por: _____ Paciente _____ Cuidador _____ Paciente e cuidador

Paciente/Participante: ____

Data: ____

1. Com que frequência você pensa sobre as seguintes atividades (por exemplo, ter dificuldades de evitar esses pensamentos ou sentir-se culpado)?

Jogar? __Nunca(0) __Raramente(1) __Às vezes(2) __Frequentemente(3)__Muito frequentemente(4)
Fazer sexo? __Nunca(0 __Raramente(1) __Às vezes(2) __Frequentemente(3)__Muito frequentemente(4)
Comprar? __Nunca(0) ___Raramente(1) __Às vezes(2)__Frequentemente(3)__Muito frequentemente(4)
Comer? __Nunca(0)___Raramente(1) __Às vezes(2)__Frequentemente(3)__Muito frequentemente(4)
Executar tarefas ou Passatempo? __Nunca(0)___Raramente(1) __Às vezes(2)__Frequentemente(3)__Muitofrequentemente(4)
Repetir atividades simples? ___Nunca(0)__Raramente(1)__Às vezes(2)___Frequentemente(3)__Muito frequentemente(4)
Tomar os remédios para a DP? __Nunca(0)__Raramente(1)___Às vezes(2) ___Frequentemente(3)__Muito frequentemente(4)

2. Você tem impulsos ou desejos de praticar as seguintes atividades abaixo que você considera que sejam excessivas ou que lhe causam angústia (inclusive, fazendo com que você se torne inquieto ou irritável quando não pode praticá-las)?

Jogar? __Nunca(0) __Raramente(1) __Às vezes(2) __Frequentemente(3)__Muito frequentemente(4)
Fazer sexo? __Nunca(0 __Raramente(1) __Às vezes(2) __Frequentemente(3)__Muito frequentemente(4)
Comprar? __Nunca(0) ___Raramente(1) __Às vezes(2)__Frequentemente(3)__Muito frequentemente(4)
Comer? __Nunca(0)___Raramente(1) __Às vezes(2)__Frequentemente(3)__Muito frequentemente(4)
Executar tarefas ou Passatempo? __Nunca(0)___Raramente(1) __Às vezes(2)__Frequentemente(3)__Muitofrequentemente(4)
Repetir atividades simples? ___Nunca(0)__Raramente(1)__Às vezes(2)___Frequentemente(3)__Muito frequentemente(4)
Tomar os remédios para a DP? __Nunca(0)__Raramente(1)___Às vezes(2) ___Frequentemente(3)__Muito frequentemente(4)

3. Você tem dificuldade em controlar as seguintes atividades detalhadas abaixo (por exemplo, essas atividades tornam-se mais frequentes com o passar do tempo, ou você tem dificuldade em reduzi-las ou interrompê-las)?

Jogar? __Nunca(0) __Raramente(1) __Às vezes(2) __Frequentemente(3)__Muito frequentemente(4)
Fazer sexo? __Nunca(0 __Raramente(1) __Às vezes(2) __Frequentemente(3)__Muito frequentemente(4)
Comprar? __Nunca(0) ___Raramente(1) __Às vezes(2)__Frequentemente(3)__Muito frequentemente(4)
Comer? __Nunca(0)___Raramente(1) __Às vezes(2)__Frequentemente(3)__Muito frequentemente(4)
Executar tarefas ou Passatempo? __Nunca(0)___Raramente(1) __Às vezes(2)__Frequentemente(3)__Muitofrequentemente(4)
Repetir atividades simples? ___Nunca(0)__Raramente(1)__Às vezes(2)___Frequentemente(3)__Muito frequentemente(4)
Tomar os remédios para a DP? __Nunca(0)__Raramente(1)___Às vezes(2) ___Frequentemente(3)__Muito frequentemente(4)

4. Você tem atitudes para continuar a ter as atividades abaixo (por exemplo, esconder o que está fazendo, mentir, colecionar coisas, pedir emprestado, acumular dívidas, roubar ou envolver-se em atos ilegais)?

Jogar? __Nunca(0) __Raramente(1) __Às vezes(2) __Frequentemente(3)__Muito frequentemente(4)
Fazer sexo? __Nunca(0 __Raramente(1) __Às vezes(2) __Frequentemente(3)__Muito frequentemente(4)
Comprar? __Nunca(0) ___Raramente(1) __Às vezes(2)__Frequentemente(3)__Muito frequentemente(4)
Comer? __Nunca(0)___Raramente(1) __Às vezes(2)__Frequentemente(3)__Muito frequentemente(4)
Executar tarefas ou Passatempo? __Nunca(0)___Raramente(1) __Às vezes(2)__Frequentemente(3)__Muitofrequentemente(4)
Repetir atividades simples? ___Nunca(0)__Raramente(1)__Às vezes(2)___Frequentemente(3)__Muito frequentemente(4)
Tomar os remédios para a DP? __Nunca(0)__Raramente(1)___Às vezes(2) ___Frequentemente(3)__Muito frequentemente(4)

Questionário para transtornos impulsivo-compulsivos na doença de Parkinson - escala de classificação (Questionnaire for Impulsive-Compulsive Disorders in Parkinson's Disease - Rating Scale, QUIP-RS)

Participante: ____

Data: ____

FICHA DE PONTUAÇÃO

**A. Jogar**	______	**(0-16)**
**B. Fazer sexo**	______	**(0-16)**
**C. Comprar**	______	**(0-16)**
**D. Comer**	______	**(0-16)**
**E. Passatempo-Atividades repetitivas compulsivas**	______	**(0-32)**
**F. Uso de remédios para a DP**	______	**(0-16)**

Pontuação Total TIC (A-D) ______ **(0-64)**

Pontuação Total QUIP-RS (A-F) ______ **(0-112)**
